# Discrimination Algorithm and Procedure of Snow Depth and Sea Ice Thickness Determination Using Measurements of the Vertical Ice Temperature Profile by the Ice-Tethered Buoys

**DOI:** 10.3390/s18124162

**Published:** 2018-11-27

**Authors:** Guangyu Zuo, Yinke Dou, Ruibo Lei

**Affiliations:** 1 College of Electrical and Power Engineering, Taiyuan University of Technology, Taiyuan 030024, China; zuoguangyu0030@link.tyut.edu.cn; 2 SOA Key Laboratory for Polar Science, Polar Research Institute of China, Shanghai 200136, China; leiruibo@pric.org.cn

**Keywords:** snow depth, ice thickness, ice-tethered buoys, temperature profiles, discrimination algorithm

## Abstract

Snow depth and sea ice thickness in the Polar Regions are significant indicators of climate change and have been measured over several decades by ice-tethered buoys. However, sea ice temperature profiles measured by ice-tethered buoys are rarely used to infer snow depth and sea ice thickness owing to the lack of automatic discrimination algorithms, restricting the use of the data for sea ice thermodynamics studies. In this study, snow depth and sea ice thickness were retrieved through the measurements of sea ice temperature profiles using discrimination algorithms of the change point and the maximum likelihood detection methods. The data measured by 50 ice-tethered buoys were used to evaluate the accuracy of the results determined by the algorithm. Influences on the seasonal sea ice thermodynamic state, vertical interval of temperature sensors on the buoys, and initial ice thickness on the estimation errors were also evaluated. The performance of the discrimination algorithm for the data from the Arctic and Antarctic regions was also compared. There were no identifiable differences between the estimation errors from the Arctic and Antarctica. Increases in both the interval of the temperature sensors and the initial ice thickness enlarged the error for the estimation of ice thickness. A procedure developed in this study strengthens the potential application of measurements from the ice-tethered buoys only with the measurements of the vertical temperature profile of the layer of snow-covered ice, but not the measurements of ice basal and surface positions using acoustic sounding.

## 1. Introduction

Sea ice is a sensitive indicator of climate change in the Polar Regions. The thickness of Arctic sea ice has declined dramatically in recent decades [1,2,3] and its accelerated melting of the sea ice in Arctic has led to changes in the ice-albedo feedback [4,5,6]. Observations of sea ice mass balance can improve the fundamental understanding of the role and sensitivity of sea ice in global climate change. In the study of sea ice mass balance, ice thickness is widely defined as the most integrated parameter for describing the sea ice conditions [7,8]. The snow on the sea ice can affect the surface albedo and heat exchange between the atmosphere and ice. The depth of snow is crucial for evaluating surface energy equilibrium and growth of sea ice, and for the retrieval algorithm of sea ice thickness using the data of satellite altimeter.

Numerical modeling is the most widely used and most effective research method to depict of the ocean–ice–atmosphere interactions in the Polar Regions [9]. The accuracy of the calculation of sea ice thickness using numerical models depends largely on assimilation of the observation of ice thickness and snow depth. Remote sensing has been widely applied to observe sea ice cover in the Polar Regions. Unlike observations of sea ice extent, monitoring the thickness of sea ice using remote sensing is considered as a more difficult task. With the development of satellite-based technologies to measure sea ice thickness, a large number of in-situ observations of snow depth and sea ice thickness are needed to be obtained for the verification and optimization algorithms for remote sensing. With the in-situ observations, sea ice cover thickness can be measured by drill–holes, upward looking sonar onboard submarines [10], electromagnetic sounding set up onboard ships or helicopters [11,12,13], and Ice Mass Balance buoys (IMBs).

In the Arctic, the Surface Heat Budget of the Arctic Ocean (SHEBA) experiment from 1997 to 1998 [14] and the Chinese National Arctic Research Expedition (CHINARE) from 2003 to 2017 [15,16] have deployed many IMBs to measure the seasonal change in sea ice mass balance. The IMBs measure snow depth, sea ice thickness, air temperature, and barometric pressure and can provide a temperature profile through the ice using a 4.5-m-long thermistor string with sensors mounted at 0.10 m intervals [17]. The acoustic sounding instruments assembled in the IMB are designed to observe snow depth and sea ice thickness. A novel and low-cost Snow and Ice Mass Balance Array (SIMBA) is designed to use digital sensor modules (spaced every 0.02 m) to build a thermistor string that measures temperature of sea ice [18]. The Polar Research Institute of China and Taiyuan University of Technology have deployed ice-tethered buoys in the Arctic Ocean since CHINARE-2014. This type of buoy (a TUT buoy) has a 4.5-m-long thermistor string, assembled using 150 thermistor sensors with an accuracy of 0.1 °C and vertical interval of 0.03 m.

Observations of snow depth and sea ice thickness using an IMB is completed by relatively expensive acoustic sounders. If the sonar sensors are damaged, the data of sea ice mass balance will not be obtained. A SIMBA can determinate the snow depth and sea ice thickness based on a heated operation mode, in which there is a significant difference in the threshold temperature of different media after heating because of the different specific heat capacities among air, snow, ice, and water. However, the automatic discrimination algorithms and the procedures of discrimination algorithm for snow depth and sea ice thickness using the measurement of vertical temperature profile of snow and ice have not been reported previously. In cases without the deployment of the acoustic sounding instruments, the vertical temperature profile through the air, snow, ice, and upper ocean can be used to discriminate snow depth and ice thickness because both the specific heats and the heat conduction coefficients are very different among air, snow, ice and water [19,20]. Generally, the vertical temperature profile measured by ice-tethered buoys has two main change points on the top and lower interfaces of the layer of snow-covered ice and the temperature profiles segmented by these two change points show remarkable differences in vertical gradient, daily amplitude, and seasonal evolution. The interface between snow and sea ice is vague because the formation of snow-ice or a slush layer cannot be distinctly identified using only the temperature profile [21,22]. The theories of change point and maximum likelihood are considered optimization methods to implement signal segmentation for identification purposes and can be used to determine the snow depth and sea ice thickness by identifying the change points of the sea ice temperature profile.

In these contexts, this paper focuses on exploring a discrimination algorithm and procedure to identify snow depth and sea ice thickness based on the methods of change point and maximum likelihood using the ice temperature profile data measured by IMBs, SIMBAs, and a TUT buoy. Section 2 describes the data measured by three ice-tethered buoys and details on the setup of the buoys. Section 3 gives a detailed description of the method of determination of the snow depth and sea ice thickness, and interfaces among air, snow, ice, and water. The discrimination algorithm based on the change point and maximum likelihood detection methods is proposed to achieve accurate detection of the top and lower interfaces of snow-covered ice. In Section 4, the temperature profiles, daily amplitude, and vertical gradients of sea ice were processed by two detection theories and the estimation accuracy was quantified by comparing the estimated results with those measured by the acoustic sounder or drilled-hole. The impacts on the estimation accuracy, including the seasonal dependence, the initial ice thickness, and the vertical interval of the temperature sensors, were analyzed and assessed in Section 5, and the performances of the discrimination algorithm for data measured in the Arctic and Antarctica were compared. Section 6 gives details on the procedure to estimate snow depth, ice surface melt, and ice thickness. The final section contains the conclusions drawn from our analysis.

## 2. Data

**Figure 1:**
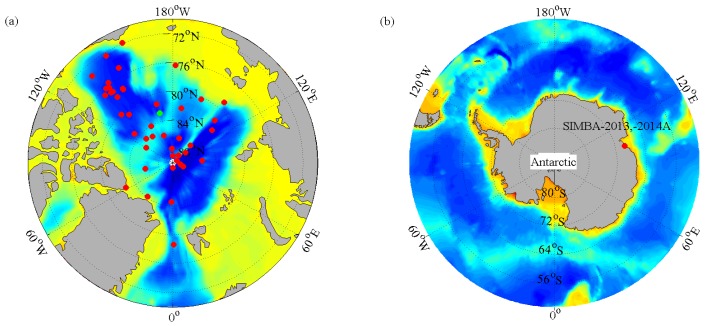
(**a**) The initial deployment positions of 47 IMB buoys (red dots) and one TUT buoy (green dot) in the Arctic from 2002 to 2016. (**b**) The initial deployment positions of two SIMBA buoys near Zhongshan Station, Antarctica in 2013 and 2014.

Three types of ice-tethered buoys were used in this study. The total number of ice-tethered buoys was 50, including 47 IMBs and one TUT buoy deployed in the Arctic, and two SIMBAs deployed in Antarctica (Figure 1 and Table 1). All the buoys are named as Type + Year + A–Z which were set according to their deployment time. For example, IMB-2008B indicates the second IMB deployed in the Arctic in 2008. Sea ice temperature profile data were measured by all ice-tethered buoys, and the temperature measurement accuracy was 0.1 °C. There are two sources of sea ice thickness and snow depth data. At the deployment of all IMB and TUT buoys, initial sea ice thickness and snow depth were observed manually. After that, the thickness of sea ice and the depth of snow were automatically observed by acoustic sounders of the buoys. For the SIMBAs, which were deployed on landfast ice near Zhongshan Station, Antarctica, ice thickness and snow depth were measured manually through drill-holes every 10–20 days during the operation of the buoys.

**Figure 2:**
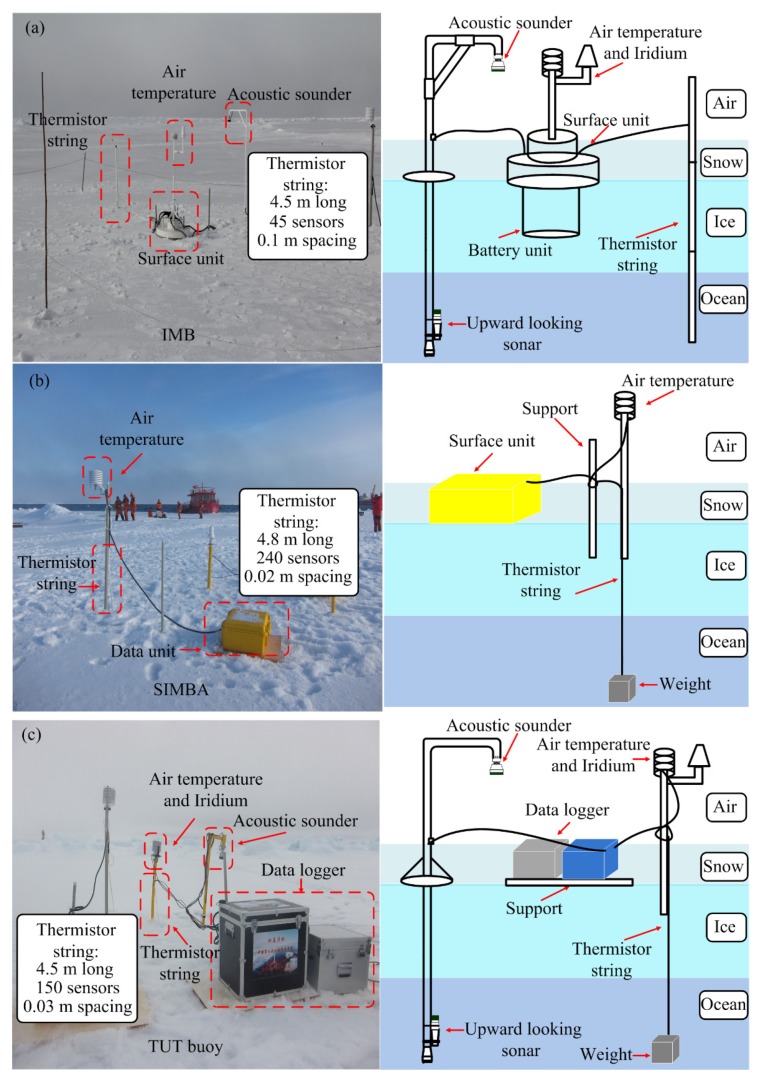
(**a**) The installation of the IMB in the Arctic Ocean during CHINARE-2016 and schematic diagram of the IMB. (**b**) The installation of the SIMBA in the Arctic Ocean during CHINARE-2016 and schematic diagram of the SIMBA. (**c**) The installation of the TUT buoy in the Arctic Ocean during CHINARE-2016 and schematic diagram of the TUT buoy.

The installations and schematic diagrams of three ice-tethered buoys are shown in Figure 2.

### 2.1. IMB Data

The IMB can be considered as one of the most reliable and widely deployed observation instruments for sea ice mass balance. A potential deployment scheme for the IMB buoy can be described as follows. The IMBs are composed of a data logger, which is in charge of data acquisition, processing and remote transmission, a sea ice mass balance observation system and a thermistor string to measure sea ice temperature profiles. The IMB uses two acoustic sensors to measure sea ice thickness and snow depth. These two acoustic rangefinder sounders are fixed on a supporting structure, which is designed to be deployed through a 0.10 m diameter ice hole. The upward looking sonar whose accuracy is 0.01 m is fixed extending from the ice hole at 3.8 m under the snow-ice interface to measure the distance from the sonar to the bottom of the ice to invert ice bottom growth and ablation. Another sensor with an accuracy of 0.001 m is installed at 1.2 m from the snow surface to measure the distance from the sensor to the top of the snow to invert snow depth. The thermistor string with a length of 4.5 m consists of three sub–strings, each of which has a length of 1.5 m. There are 45 temperature probes evenly arranged on the temperature string at intervals of 0.01 m. And the IMB data are obtained from Ice Mass Balance (IMB) Buoy Program. Here, we used 47 IMB buoys deployed from 2002 to 2014. There were 11 buoys completing stable operation for more than one year and a buoy running for more than two years (25 months).

### 2.2. SIMBA Data

SIMBAs are now widely deployed in the Arctic and Antarctica. The SIMBAs were assembled by the Scottish Association for Marine Science and modified by the Polar Research Institute of China. A SIMBA thermistor string had 240 temperature probes at intervals of 0.02 m. The data for the temperature profile of landfast ice in Antarctica near Zhongshan Station used in this paper were measured by two SIMBAs (SIMBA-2013A and SIMBA-2014A) deployed in 15 May 2013 and 13 May 2014, respectively. The observations of SIMBA-2013A and SIMBA-2014A continued until 27 November 2013 and 23 November 2014, respectively. The SIMBAs used in this study had no sonar, thus the snow depth and ice thickness of the SIMBAs were measured by drilled-hole ever 10 to 20 days during the operation of the buoys.

### 2.3. TUT Buoy Data

The Polar Research Institute of China and Taiyuan University of Technology have started to develop and deploy a new type of ice-tethered buoy (TUT buoy) since CHINARE-2014 in the Arctic Ocean. The TUT buoy uses digital temperature sensor chips with a resolution of 0.0625 °C and an accuracy of 0.1 °C. A 4.5-m-long thermistor string is linked by 15 flexible printed circuit boards (FPCBs) with a length of 0.3 m. Two acoustic rangefinder sounders (Campbell SR50A and Teledyne-Benthos PSA916, respectively) were used to measure snow depth and ice thickness. Based on a 32-bit low-power microcontroller, we designed the data acquisition instrument and the iridium satellite data transmission system, which allowed data to be sent back to the lab computer in real time. The TUT buoy (TUT-2016A) used in this paper was deployed over multiyear ice in the Arctic on 9 August 2016 during the CHINARE-2016. The measurements, including snow depth, ice thickness, and sea ice temperature profiles of TUT-2016A, lasted from 9 August 2016 to 11 July 2017, with the data sampling interval of one hour.

## 3. Methods for Data Processing

### 3.1. Determination of the Sea Ice Thickness/Snow Depth and Interfaces Among Air, Snow, Ice, and Water

The thickness of sea ice and the depth of snow can be calculated by determination of the media interfaces: top interface of air-snow or air-ice (when the snow has melted completely), snow-ice interface and the lower interface of ice-water. The interfaces can be derived from the temperature profiles observed by the ice-tethered buoys. Through identifying changes in the positions of interfaces, snow depth and sea ice thickness can be obtained.

The snow-ice interface is considered to be constant except for slush formation associated with flooding [22]. This phenomenon can be observed by the heating mode of the SIMBA. In this paper, IMB data with a temperature sensor interval of 0.10 m was mainly analyzed. The interval of TUT buoy is 0.03 m, but similar to the IMB, the TUT buoy does not have a heating mode as SIMBA to identify the interface of snow-ice and observe the evolution of snow to ice. Thus, the algorithm to identify the snow-ice interface could not be thoroughly considered and we did not develop the algorithm to solve this problem in this paper. Hereafter, we simply assumed the position of the snow-ice interface was constant.

The interfaces between air and snow or ice, and between ice and water were identified using the temperature profiles, the daily amplitude, which are equal to the differences between the maximum and minimum values of the daily temperature profiles of each sensor, and the vertical temperature gradients. The temperature profile, daily amplitude of temperature profiles and the vertical gradients of the temperature were widely used to estimate the interface between air and snow (or ice) [16,21,22]. The vertical gradient in air was different from in snow and ice. The daily amplitude of temperature profile was larger in air than in snow. The temperature profiles were measured by the buoys, and the daily amplitude of temperature profiles and the vertical temperature gradients were calculated from the temperature profile.

#### 3.1.1. Interface between Air and Snow or Sea Ice

The evolution of snow depth over sea ice may be affected by synoptic processes phenomena such as storms, snowfall, and sleet, or due to melting caused by solar radiation. Thus, the temporal fluctuations of snow depth are much larger than for sea ice thickness. The daily amplitude of temperature profiles and the vertical gradients of temperature were examined because, in some cases, it was difficult to accurately distinguish the top interface by using the measurements of the temperature alone. From the data of the daily amplitude of temperature profiles and the vertical gradients of temperature, we checked the corresponding values one by one from the top of the temperature profiles to find the change points.

**Figure 3:**
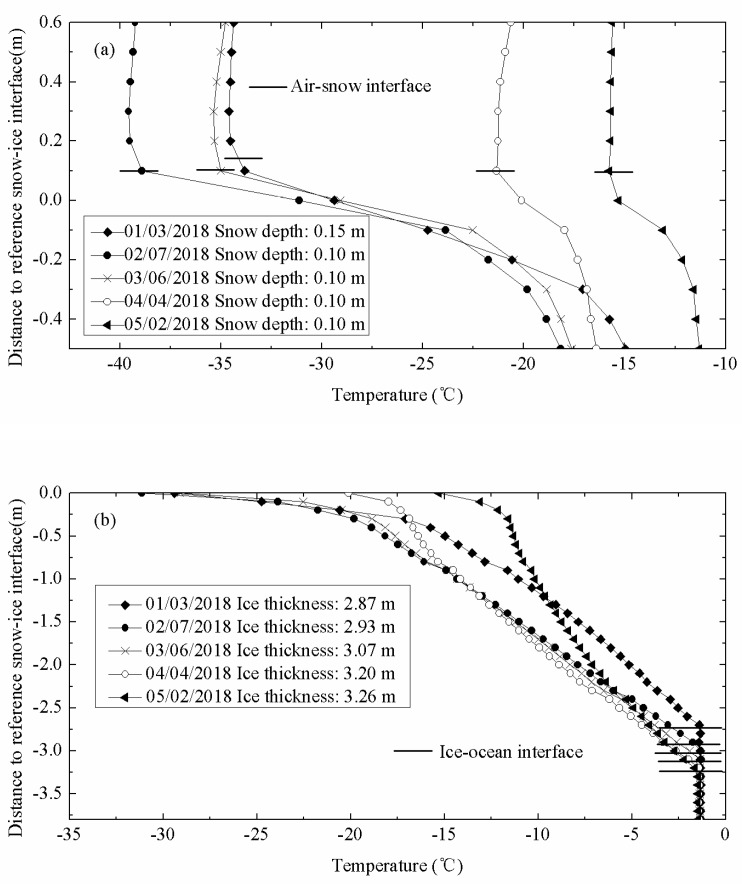

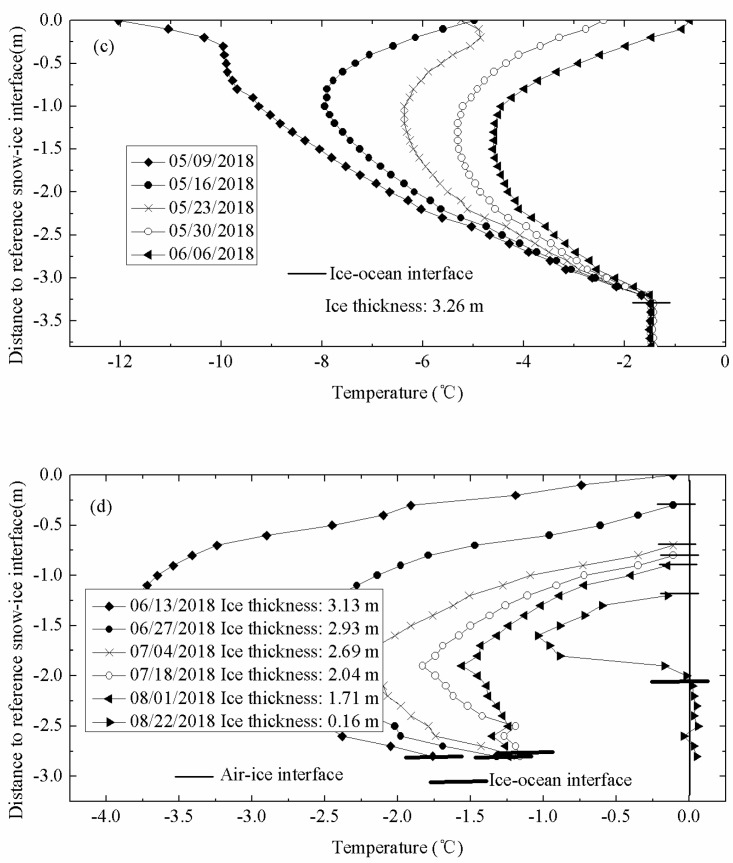
The vertical temperature profiles (resolution of 0.10 m) through air, snow, sea ice, and upper ocean measured by IMB-2007E. (**a**) Typical top profiles (12 points) were obtained from 3 January to 2 May (freezing period). (**b**) Typical profiles under the initial ice surface were obtained from 3 January to 2 May (freezing period). (**c**) Typical profiles under the top interface were obtained from 9 May to 6 June (warming period). (**d**) Typical profiles under the initial ice surface were obtained from 13 June to 22 August (melting period).

Figure 3 shows the vertical temperature profiles through air, snow, sea ice and upper-ocean measured by IMB-2007E. IMB-2007E was deployed on 16 August 2007 in the Arctic sea ice (78.9° N, 139.9° W) and drifted until 16 October 2008. The depth of snow and thickness of ice were measured by sonar. 

In winter or sea ice growth period, sea ice was covered by deep snow, so the air-snow interface was considered as the top interface. As shown in Figure 3a, the obvious inflection point of the temperature profiles occurred at the snow-ice interface, with a near constant temperature gradient above this interface. During this period, the internal temperature profile through the sea ice was basically linear (Figure 3b). In the warming period, the snow cover may sublimate or melt. When the ice cover reached a thermodynamic balance, the ice thickness increased to the maximum value for the entire ice season (Figure 3c).

**Figure 4:**
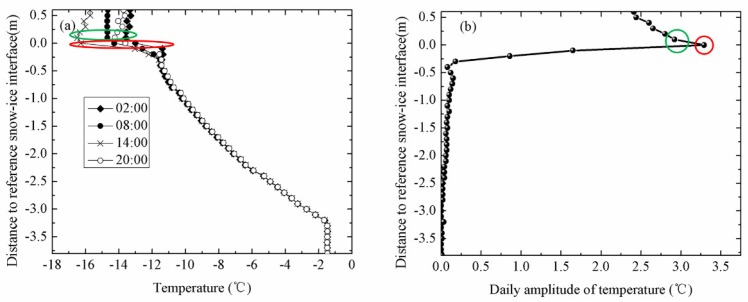
(**a**) Temperature profiles of IMB-2007E on 2 May and (**b**) the daily amplitudes of temperature. The green circle is the air-snow interface and the red circle is the snow-ice interface.

The temperature profile of sea ice at the end of the warming period was C-shaped. Correspondingly, the temperatures measured by the sensors exposed to the air were above 0 °C. The intersection of the top temperature profile with 0 °C was identified as air-ice interface (Figure 3d). The daily amplitude of temperature profile of sea ice can be described as follows:

(1) $$D(i)=T_{\max}(i)-T_{\min}(i)$$

where *D*(*i*) is the daily amplitude of a certain point on the temperature profile; *T_max_*(*i*) and *T_min_*(*i*) are the maximum temperature and minimum temperature at a certain time of a day, respectively. The measurements at the top of temperature profiles had significant daily amplitudes of about 4.5 °C, as shown in Figure 4. However, the bottom of temperature profiles had minor daily changes in our study. By examining the sharp change point of daily temperature profiles, the top interfaces can easily be determined. The temperature profile of snow remained linear in winter, but its vertical gradients of temperature differ from the sea ice because the different coefficients of heat conduction among air, snow, and ice [16,21,22].

**Figure 5:**
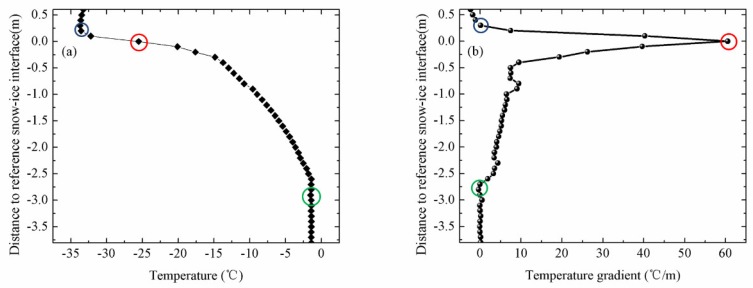
(**a**) Temperature profile of IMB-2007E on 20 December and (**b**) vertical gradients of temperature profile. The blue circle is the air-snow interface. The red circle is the snow-ice interface. The green circle is the ice-ocean interface.

The temperature gradient of temperature profile can be described as follows [16,21,22]:

(2) $$G=\frac{\partial T}{\partial Z}$$

where *G* is the temperature gradient of the temperature profile; *T* is the temperature measured by buoys; z is the vertical coordinate. As shown in Figure 5, the interface between air and snow can be estimated from the vertical gradients of temperature by seeking the sharp change point of temperature gradients.

#### 3.1.2. Interface between Ice and Ocean

To identify the lower ice interface (ice-ocean interface), temperature profiles for the lower ice layer were obtained from some thermistors near the bottom of the sea ice. The seawater temperature was determined using the lower five thermistors, which generally had a negligible temperature gradient from the bottom of the sea ice. The points where the temperature profile of the lower ice layer intersected the ocean temperature were regarded as the ice-ocean interface (Figure 3b). The ice-ocean interface determined by the method of seeking described above had a good accuracy in winter or sea ice growth period. This method became unreliable in summer, especially in ice melting period when the temperature gradient across the lower interface weakened. In summer, the temperature profile of sea ice became nonlinear with a C-shaped curve. Then the lower ice interface was determined from the obvious inflection point in the vertical C-shaped ice temperature profile (Figure 3d). In winter, temperature profile of sea ice remained linear and temperature of the basal ice layer was colder than the upper ocean. There will be a sharp inflection point occurring at the interface. Thus, ice-ocean interface can be estimated from the vertical gradients of sea ice temperature profile.

### 3.2. Change Point Detection Method

In Section 3.1, we introduced the principle of distinguishing the top and lower interfaces based on the sea ice temperature profile, the daily amplitude of temperature profiles, and the vertical temperature gradients. The calculation accuracy of sea ice thickness and snow depth depends on the accuracy and consistency of interface discrimination.

An off-line change point detection method was used to obtain the optimal estimation of inflection points derived from data of ice-tethered buoys, which were used to identify the top and lower ice interfaces. We assumed that there were N points in the sea ice temperature profiles, so that the sea ice temperature profile is described as follows:

(3) $$\left\{ \begin{array}{l} S_{k}=T_{1}+e_{k},1\leq k<t_{1}\\ S_{k}=T_{2}+e_{k},t_{1}\leq k<t_{2}\\ S_{k}=T_{3}+e_{k},t_{2}\leq k\leq N \end{array}\right.$$

where *T*_1_ is the temperature of air or snow; *T*_2_ is the temperature of sea ice; *T*_3_ is the temperature of ocean. In this study, the vertical gradients of temperature and daily amplitude of temperature profiles were also used to distinguish snow depth and ice thickness. For these estimations, *T*_1_, *T*_2_, *T*_3_ represented the vertical gradients of temperature or daily amplitude of temperature profiles, respectively. *S_k_* was the temperature value from the temperature profile. *t*_1_ was the location of the ice-snow interface, *t*_2_ was the location of the ice-ocean interface, and *e_k_* was the noise in the data of sea ice temperature profile. The detection of multiple change points can be accomplished with the single change point detection method, the intent of which is to estimate of the change points.

The principle of the least-squares change point estimation is to take a change point to minimize the sum of the squares of the segment errors between the two subgroups where the change point is the demarcation point. Thus, this method can be applied to the discrimination of the top and lower interfaces. The target formula can be described as follows:

(4) $$I_{top}=\arg\underset{1\leq m\leq C}{\min}\left\{\sum_{i=1}^{m-1}{\left[S_{i}-E_{1}\left(m\right)\right]}^{2}+\sum_{i=m}^{C}{\left[S_{i}-E_{2}\left(m\right)\right]}^{2}\right\}$$

(5) $$I_{lower}=\arg\underset{C+1\leq m\leq N}{\min}\left\{\sum_{i=C+1}^{m-1}{\left[S_{i}-E_{3}\left(m\right)\right]}^{2}+\sum_{i=m}^{N}{\left[S_{i}-E_{4}\left(m\right)\right]}^{2}\right\}$$

where *I_top_* is the estimate of the top interface and *I_lower_* is the estimate of the lower interface. *C* stands for a point in the sequence number of data cluster. *E*_1_(*m*), *E*_2_(*m*), *E*_3_(*m*) and *E*_4_(*m*) are both segmented mean estimations of the data cluster:

(6) $$E_{1}\left(m\right)=\frac{1}{m-1}\sum_{i=1}^{m-1}S_{i}$$

(7) $$E_{2}\left(m\right)=\frac{1}{C-m+1}\sum_{i=m}^{C}S_{i}$$

(8) $$E_{3}\left(m\right)=\frac{1}{m-C+1}\sum_{i=C+1}^{m}S_{i}$$

(9) $$E_{4}\left(m\right)=\frac{1}{N-m+1}\sum_{i=m+1}^{N}S_{i}$$

We introduced the least-absolute deviations in the estimation of the change point. The principle of this estimation method is to define a change point to minimize the absolute value of the segment error, for which the least-absolute deviations is more robust to outliers than the least-squares method on the handling of errors. The least-squares change point estimation brings in larger errors, which are often the outliers in the measurements and may lead to false results of change point estimation. The least-absolute deviations method can effectively reduce the effect. The least-absolute deviations estimation of change point can be described as follows:

(10) $$I_{top}=\arg\underset{1\leq m\leq C}{\min}\left\{\sum_{i=1}^{m-1}\left|S_{i}-E_{1}\left(m\right)\right|+\sum_{i=m}^{C}\left|S_{i}-E_{2}\left(m\right)\right|\right\}$$

(11) $$I_{lower}=\arg\underset{C+1\leq m\leq N}{\min}\left\{\sum_{i=C+1}^{m-1}\left|S_{i}-E_{3}\left(m\right)\right|+\sum_{i=m}^{N}\left|S_{i}-E_{4}\left(m\right)\right|\right\}$$

In the change point detection method, we propose a selection algorithm for change point detection to improve the accuracy of inflection point detection. The selection algorithm of two change points is described as follows:

- Input: The sea ice temperature profile or the daily amplitude of temperature profiles or the vertical temperature gradients *S_k_* (*k* = 1, 2, 3, …, *N*).
- Step 1: For the input parameters *S_k_* (*k* = 1, 2, 3, …, *N*), we use the Equation (10) to get a change point *C*_0_ and two new profiles *S_k_* (*k* = 1, 2, 3, …, *C*_0_) and *S_k_* (*k* = *C*_0_ + 1, *C*_0_ + 2, *C*_0_ + 3, …, *N*).
- Step 2: We use the Equation (10) to process *S_k_* (*k* = 1, 2, 3, …, *C*_0_) to get a change point *C*_11_. Then a change point *C*_12_ detected by the Equation (11) based on *S_k_* (*k = C*_11_ + 1, *C*_11_ + 2, *C*_11_ + 3, …, *N*). The error of change point estimation is *E*_1_.
- Step 3: For the profile *S_k_* (*k = C*_0_ + 1, *C*_0_ + 2, *C*_0_ + 3, …, *N*), we use the Equation (11) to get a change point *C*_22_. A new change point *C*_21_ detected by the Equation (10) based on *S_k_* (*k* = 1, 2, 3, …, *C*_22_). The error of change point estimation is *E*_2_.
- Step 4: If *E*_1_ < *E*_2_, *C*_11_ and *C*_12_ are two change points representing the top and lower interfaces. Otherwise, *C*_21_ and *C*_22_ are two change points.
- Output: Two change points and calculated ice thickness and snow depth.

### 3.3. Maximum Likelihood Detection Method

Here, we used an off-line mean change point detection method for a normal distribution based on the maximum likelihood method for the optimal estimation of the change point position. First, we discuss the situation of single change points, and then expand to the detection method of multiple points. *S_k_* was the temperature value from the temperature profile and *t* was the only change point of the profile:

(12) $$\left\{ \begin{array}{l} S_{k}=T_{1}+e_{k},1\leq k<t\\ S_{k}=T_{2}+e_{k},t\leq k\leq N \end{array}\right.$$

The noise of this profile can be de described as follows:

(13) $$\left\{ \begin{array}{l} e_{k}∼N\left(0,\sigma_{1}{}^{2}\right),1\leq k<t\\ e_{k}∼N\left(0,\sigma_{2}{}^{2}\right),t\leq k\leq N \end{array}\right.$$

If the noise is Gaussian white noise, the likelihood function is described as follows:

(14) $$L\left(t\right)=\prod_{i=1}^{N}f\left(x_{i}\right)={\left(2\pi \right)}^{\frac{-n}{2}}\cdot \sigma_{1}{}^{-\left(t-1\right)}\cdot \sigma_{2}{}^{-\left(n-t+1\right)}\cdot \exp\left\{\frac{-\sum_{i=1}^{t-1}{\left(S_{i}-E_{1}\right)}^{2}}{2\sigma_{1}{}^{2}}\right\}\cdot \left\{\frac{-\sum_{i=t}^{N}{\left(S_{i}-E_{2}\right)}^{2}}{2\sigma_{2}{}^{2}}\right\}$$

The parameters *E*_1_, *E*_2_, *σ*_1_ and *σ*_2_ are both uncertain. To acquire the likelihood maximum, we calculated the derivative of *E*_1_ and *E*_2_, respectively and made the derivative equals zero. Then we get a set of equations:

(15) $$E_{1}=\frac{1}{t-1}\sum_{i=1}^{t-1}S_{i}$$

(16) $$E_{2}=\frac{1}{n-t+1}\sum_{i=t}^{N}S_{i}$$

(17) $$\sigma_{1}{}^{2}=\frac{1}{t-1}\sum_{i=1}^{t-1}{\left(S_{i}-E_{1}\right)}^{2}$$

(18) $$\sigma_{2}{}^{2}=\frac{1}{n-t+1}\sum_{i=t}^{N}{\left(S_{i}-E_{2}\right)}^{2}$$

We plugged the results of Equations (15)–(18) to simplify the Equation (14), and we can get a simplified equation as follows:

(19) $$L\left(t\right)=2\pi^{\frac{-N}{2}}\cdot e^{{}^{\frac{-N}{2}}}{\left\{\frac{\sum_{i=1}^{t-1}{\left(S_{i}-E_{1}\right)}^{2}}{t-1}\right\}}^{\frac{-\left(t-1\right)}{2}}\cdot {\left\{\frac{\sum_{i=t}^{N}{\left(S_{i}-E_{2}\right)}^{2}}{N-t+1}\right\}}^{\frac{-\left(N-t+1\right)}{2}}$$

The maximum of the Equation (19) is equivalent to the minimum value of the following log-likelihood, which is the maximum likelihood estimation of the change point of the normal distribution:

(20) $$G\left(t\right)={\left[\frac{\sum_{i=1}^{t-1}{\left(S_{i}-E_{1}\right)}^{2}}{t-1}\right]}^{\frac{-\left(t-1\right)}{2}}\cdot {\left[\frac{\sum_{i=t}^{N}{\left(S_{i}-E_{2}\right)}^{2}}{N-t+1}\right]}^{\frac{-\left(N-t+1\right)}{2}}$$

After applying a logarithm to Equation (20), a new equation was obtained:

(21) $$Ln\left(G\left(t\right)\right)=\frac{t-1}{2}Ln\sigma_{1}{}^{2}+\frac{N-t+1}{2}Ln\sigma_{2}{}^{2}$$

The *t* that makes the minimum value of the upper form is the maximum likelihood estimation of the position of the change point. However, in the calculation of sea ice thickness and snow depth, two change points are used. Here, *S_k_* are the temperature values from the temperature profile and have two change points of *t*_1_ and *t*_2_. A set of 4D vectors *T* (0, *t*_1_, *t*_2_, *N*) is introduced. The criterion of change point estimation by maximum likelihood method is that seeking out a group of *T* (0, *t*_1_, *t*_2_, *N*) to make the next equation have the minimum value of solution:

(22) $$L=\sum_{i=1}^{3}\frac{T\left(i+1\right)-T\left(i\right)+1}{2}Ln\sigma_{i}{}^{2}$$

(23) $$\sigma_{i}{}^{2}=\frac{1}{T\left(i+1\right)-T\left(i\right)+1}\sum_{j=T\left(i\right)+1}^{T\left(i+1\right)}{\left(S_{j}-E_{i}\right)}^{2}$$

(24) $$E_{i}=\frac{1}{T\left(i+1\right)-T\left(i\right)+1}\sum_{j=T\left(i\right)+1}^{T\left(i+1\right)}S_{j}$$

## 4. Estimation Error

### 4.1. Estimation Error of the Change Point Detection Method

#### 4.1.1. The Results Calculated Using Ice Temperature Profile

We combined the calculated top and lower interfaces to obtain the ice thickness and compared with “actual” ice thickness. The “actual” ice thickness of IMBs and TUT was measured by acoustic sounders and the “actual” ice thickness of SIMBAs was measured by drilled holes. The maximum of average error of sea ice thickness was 0.0948 m in IMB-2009D, whereas the minimum of average error of sea ice thickness was 0.0205 m in SIMBA-2013A. The estimation error of the entire samples of sea ice buoys is distributed in a relatively narrow range. We set the estimation error within 0–0.06 m as a small error, denoted as SE, which were 92% of the total samples. In detail, the errors in SE were mainly within 0.02–0.03 m (54%). There were 19 groups with errors ranging from 0.03–0.06 m, and there were five groups with sea ice thickness errors of 0.03–0.04 m. The number of buoys with errors of 0.04–0.05 m and 0.05–0.06 m were seven groups, respectively. The errors of sea ice thickness within 0.07–0.09 m were described as a medium error affected 6% of the 50 buoys, denoted as ME. The sea ice thickness errors of more than 0.09 m can be considered as a large error and was 6%, denoted as LE. The maximum error of snow depth was 0.092 m obtained for IMB-2011C. The minimum error of snow depth was 0.017 m in SIMBA-2013A. There were three groups with errors of 0.01–0.02 m, 23 groups with errors of 0.02–0.03 m, six groups with errors of 0.03–0.04 m, 10 groups with errors of 0.04–0.05 m, four groups with errors of 0.05–0.06 m, one group with errors of 0.06–0.07 m, 0.07–0.08 m, 0.08–0.09 m, and above 0.09 m, respectively.

#### 4.1.2. The Results Calculated Using Daily Amplitude of Temperature Profiles

The change point detection method can be simple to determine the top interface using daily amplitude of temperature profiles. However, sea ice basal interface cannot reliably be estimated using the daily change of temperature profiles. The range of estimation error of snow depth was small. The maximum (minimum) average error of snow depth was 0.105 m (0.023 m) obtained for IMB-2011C (SIMBA-2013A).

#### 4.1.3. The Results Calculated Using Vertical Gradients of Temperature

**Figure 6:**
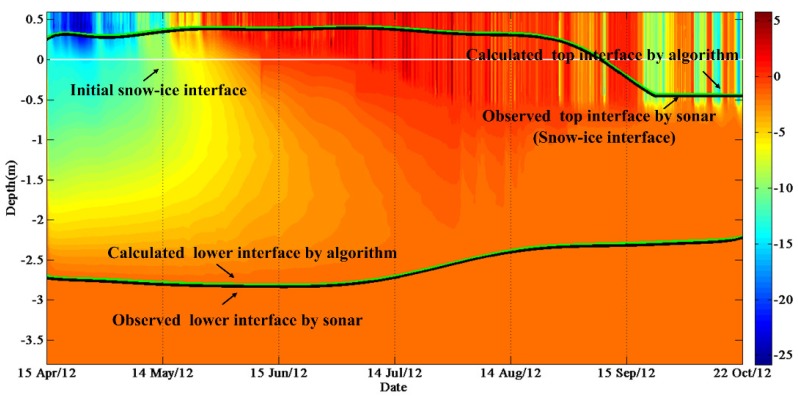
Sea ice temperature contours from 15 April to 23 October 2012 measured by IMB-2012B. The black lines are observed top and lower interfaces. The green lines are calculated top and lower interfaces. The white lines are initial snow-ice interfaces.

The calculated and observed ice thicknesses were consistent (Figure 6). For example, the buoy named IMB-2012B was deployed in the Arctic with initial ice thickness 2.72 m on 15 April, 2012. The sea ice thickness at this site has quickly reached its maximum value (2.84 m) on 12 June, and continued to decrease until 16 October when the ice thickness reached its minimum value (2.26 m).

The maximum error of sea ice thickness was 0.092 m in IMB-2009D. The minimum error of sea ice thickness was 0.017 m in SIMBA-2013A. There were two of the total buoys in ME, and one buoy in LE. The maximum (minimum) error of snow depth was 0.098 m (0.019 m) obtained for the IMB-2011C (SIMBA-2013A).

### 4.2. Estimation Error of the Maximum Likelihood Detection Method

#### 4.2.1. The Results Calculated Using Ice Temperature Profile

**Figure 7:**
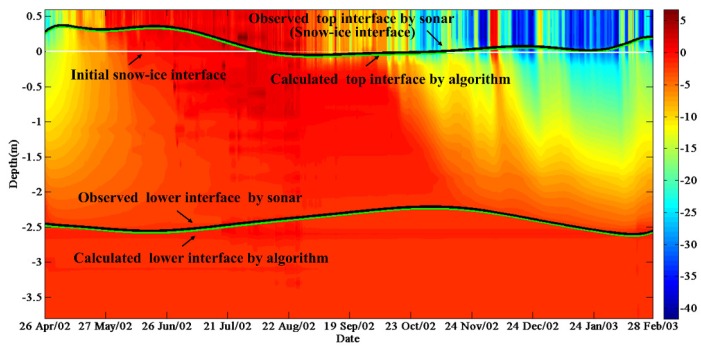
Sea ice temperature contours from 26 April 2002 to 23 February 2003 measured by IMB-2002A. The black lines are observed top and lower interfaces. The green lines are calculated top and lower interfaces. The white lines are initial snow-ice interfaces.

Temperature profiles were used to calculate top and lower interfaces of sea ice through the maximum likelihood detection method (Figure 7). The floe which IMB-2002A was deployed had initial ice thickness of 2.46 m on 26 April 2002. The entire ice season of this site included two ice thickness peaks which the earliest peak of sea ice thickness (2.51 m) occurred on 14 July. The sea ice thickness of this site reached its minimum value of 2.20 m and then entered the ice growth season until 15 February 2003, when the ice thickness reached its maximum of 2.59 m. The maximum (minimum) value of average error of sea ice thickness was 0.088 m (0.017 m) obtained for IMB-2009D (SIMBA-2013A).

The maximum (minimum) error of snow depth was 0.096 m (0.019 m) obtained for IMB-2011C (IMB-2003C). There were two groups with errors of 0.01–0.02 m, 17 groups with errors of 0.02–0.03 m, 12 groups with errors of 0.03–0.04 m, 10 groups with errors of 0.04–0.05 m, four groups with errors of 0.05–0.06 m, and one group with errors of 0.06–0.07 m, two groups with errors of 0.07–0.08 m, one group with errors of 0.08–0.09 m, one group with errors of above 0.09 m, respectively.

#### 4.2.2. The Results Calculated Using Daily Amplitude of Temperature Profile

The maximum error of snow depth using daily amplitude of temperature profiles was 0.093 m obtained for the IMB-2011C, and the minimum error of snow depth was 0.019 m obtained from two buoys of IMB-2003C in Arctic and SIMBA-2013A in the Antarctica. The range of estimation error of snow depth is small, which there were 46 buoys in SE, three buoys in ME, and one buoy in LE.

#### 4.2.3. The Results Calculated Using Vertical Gradients of Temperature

The maximum error of sea ice thickness was 0.094 m in IMB-2009D. The minimum error of sea ice thickness was 0.014 m in IMB-2012J. There were two of the total buoys in ME, and one buoy in LE. The maximum error of snow depth was 0.097 m in IMB-2011C. The minimum error of snow depth was 0.016 m in SIMBA-2013A.

### 4.3. Statistical Errors of the Two Methods

In general, all the calculated average errors compared to the observed values of ice thickness obtained by the methods of change point and maximum likelihood are within a range of 0–0.11 m. After we used two methods to process the data of temperature profile, daily amplitude of temperature profile and vertical gradients of temperature profile, the maximum average errors of ice thickness occurred in IMB-2009D. The minimum average errors of ice thickness occurred in SIMBA-2013A (which appeared five times) and IMB-2012J (it appeared once). Correspondingly, all the calculated average errors of snow depth were 0.016–0.105 m. The maximum average errors of snow depth occurred in IMB-2011C. The minimum average errors of snow depth occurred in SIMBA-2013A (it appeared five times) and IMB-2003C (it appeared two times).

**Figure 8:**
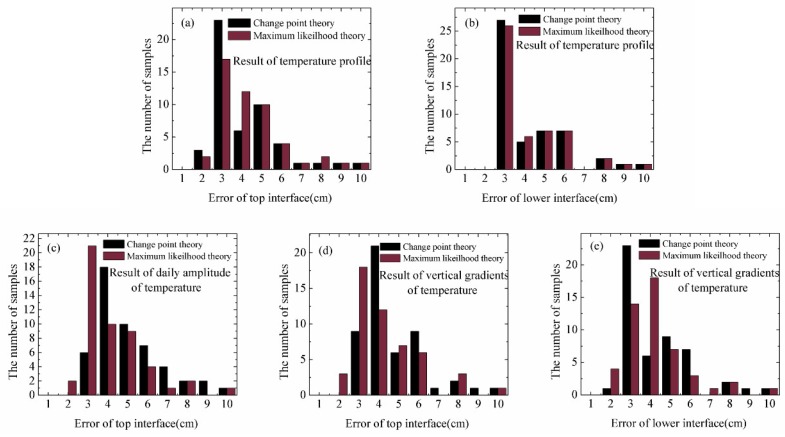
The statistical errors of snow depth (top interface) and ice thickness (lower interface) of the change point detection method and maximum likelihood detection method using temperature profiles (**a**,**b**), daily change of temperature (**c**), and vertical gradients of temperature (**d**,**e**).

In Figure 8 we show the statistical results of error of snow depth (top interface) and ice thickness (lower interface) using the methods of change point detection method and maximum likelihood detection method for the data measured by 50 ice-tethered buoys.

In Figure 8a, the histogram has peaks of error of 0.02–0.03 m and 0.03–0.04 m. Change point theory had better performance with 14% more groups appearing in the smaller error range (<0.03 m) compared to the maximum likelihood detection method. However, the distribution trends of error in the top interface presented by the two methods are basically the same.

The results of calculating ice thickness using the temperature profiles by two methods are shown in Figure 8b. The difference only occurred in the intervals of 0.02–0.03 m and 0.03–0.04 m. Other sets of results remained highly consistent.

In Figure 8c, maximum likelihood detection method can reduce estimated errors of snow depth using daily amplitude of temperature compared to the change point method. There were 10% errors of 50 buoys in SE using maximum likelihood detection method more than the other method. As results of change point theory, estimated errors of snow depth was larger than 0.02 m.

The calculation of top interfaces using maximum likelihood method was closer to the observed values compared to that obtained using the change point method, with average errors of 0.038 m to 0.043 m, respectively, as shown in Figure 8d.

The results of calculating ice thickness using the vertical gradients of temperature by two methods are shown in Figure 8e. Two methods had comparable average error of ice thickness (0.038 m and 0.037 m). Errors calculated by the maximum likelihood detection method were 43% appearing in 0.02–0.03 m, while the probability was 28% for the change point detection method.

The maximum and minimum errors of ice thickness and snow depth calculated by the two methods based on three parameters are shown in Table 2. It indicates that the performance of the discrimination of ice thickness by the maximum likelihood theory based on temperature profile and the discrimination of snow depth by the change point theory using the same parameter are better than other combinations.

## 5. Factors Influencing on the Estimation Error

### 5.1. Seasonal Dependence of Estimation Error

**Figure 9:**
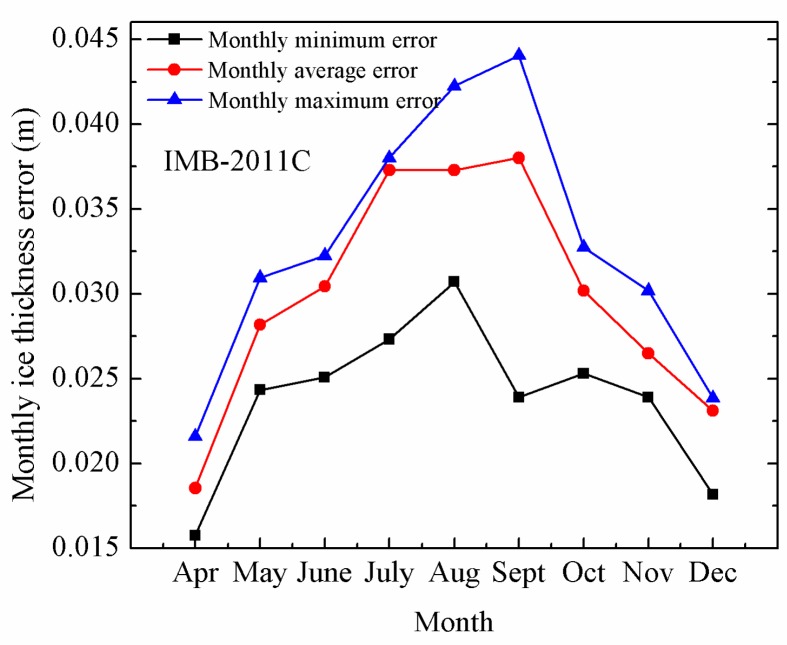
The monthly maximum error (blue line), minimum error (black line) and average error (red line) of sea ice thickness derived from the measurements of IMB-2011C, respectively.

As shown in Figure 9, IMB-2011C was an IMB buoy deployed in the Arctic in late April and the maximum (minimum) value of the average deviation of sea ice thickness was 0.038 m (0.018 m) obtained in September (April) 2011.

**Figure 10:**
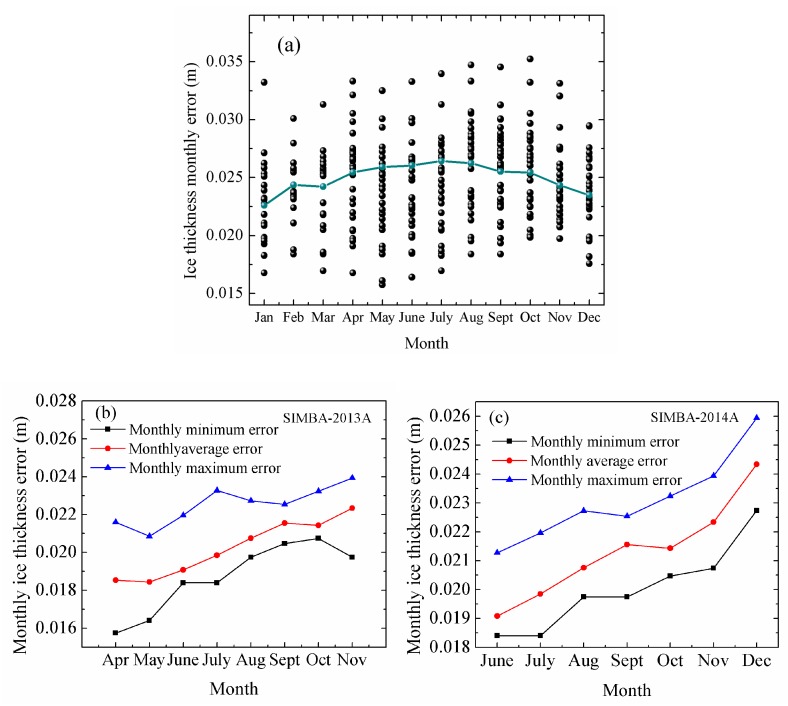
(**a**) Monthly error of ice thickness for Arctic sea ice. (**b**,**c**) Monthly error of ice thickness for Antarctic sea ice. Dark blue line represents average monthly error.

We showed 48 buoys with monthly ice thickness error in the Arctic and two buoys in Antarctica. The maximum value of average error was 0.026 m obtained in July and the minimum value of average error was 0.022 m obtained in January in the Arctic (Figure 10a). For SIMBA-2013A, the maximum value of average error was 0.023 m in November and the minimum value of average error was 0.019 m in April (Figure 10b). For SIMBA-2014A, the maximum value of average error was 0.024 m in December and the minimum value of average error was 0.019 m in June (Figure 10c).

In summer, sea ice was at the melting period, the temperature of the upper ocean was colder than the ice cover, and a large number of bubbles and pores appeared in the middle layer of sea ice. At this time, sea ice temperatures may be in an isothermal state. And in such state, the calculated sea ice thickness will have larger error. In winter, when sea ice was in its freezing period, the sea ice internal temperature gradient was established very well, and the difference of temperature between ice and upper ocean was more obvious. Thus, the calculated sea ice thickness had a smaller error.

### 5.2. The Influence of the Vertical Interval of Temperature Sensors

**Figure 11:**
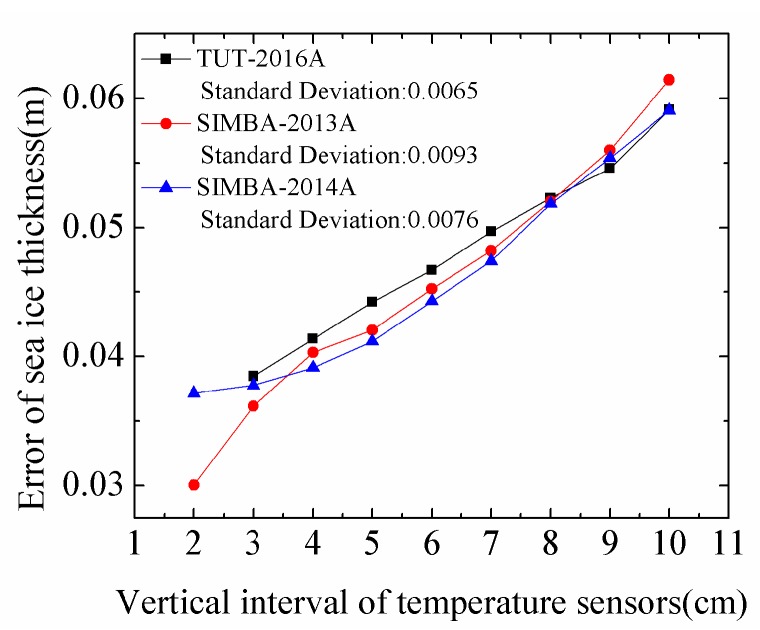
The estimated errors of ice thickness against the resampled interval of the ice temperature profile. TUT-2016A, SIMBA-2013A, SIMBA-2014A have initial vertical intervals of 0.03 m and 0.02 m, respectively.

When we used the discrimination algorithm to calculate the sea ice thickness, the temperature sensor intervals on the thermistor strings influenced the accuracy of sea ice thickness. The thermistor strings of IMBs have 45 temperature sensors equidistantly distributed with spacing of 0.10 m. The SIMBAs have 240 temperature sensors with 0.02 m interval on a flexible chain. TUT buoy has 150 temperature sensors and its temperature sensors interval is 0.03 m. We selected a TUT and two SIMBA buoys to analyze the effect of vertical spacing of temperature sensors on the calculation of ice thickness (Figure 11) and resampled the temperature profile data from 0.02 m to 0.10 m.

As shown in Figure 11, the estimation error of sea ice thickness increases as the vertical interval of ice temperature increases and the maximum error is at interval of 0.10 m. This is because the larger vertical interval will bring in larger uncertainties for the measurements of sea ice temperature and for the internal temperature gradient of sea ice.

### 5.3. The Influence of Initial Ice Thickness

**Figure 12:**
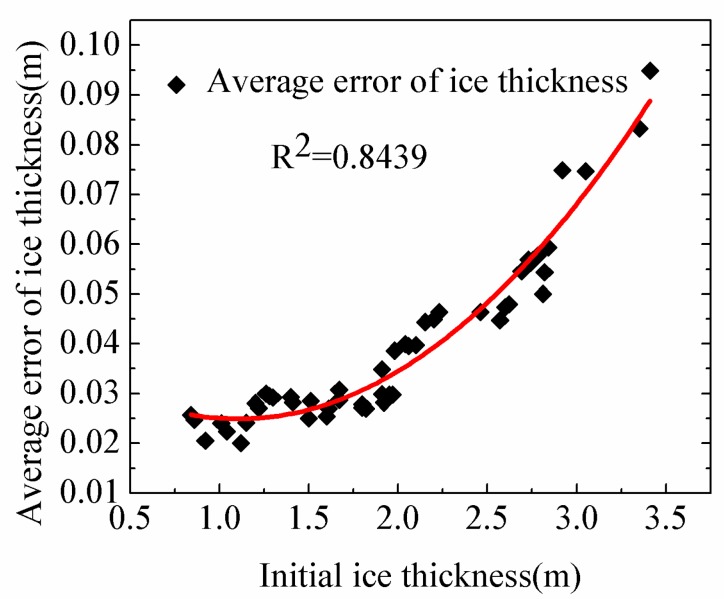
The average estimation error of sea ice thickness against the initial sea ice thickness. The red line is the fitted curve line.

Typically, ice floes in the Arctic and Antarctic with thicker initial ice thickness would have smaller growth rate and earlier onset of ice melt. Figure 12 shows the relationship between the initial sea ice thickness and the average sea ice thickness error, suggesting that the buoys with the smaller initial ice thickness would have the smaller estimation error of sea ice thickness, which is likely to attributed to the fact that the vertical gradient of temperature at the basal layer is hard to be established and still ambiguous even in the early freezing season for thicker ice thickness.

The correlation between average error of sea ice thickness and initial ice thickness is clear. In the future, as the thinning of Arctic sea ice cover, our algorithm will become more reliable.

### 5.4. Comparison between the Results Observed by the Buoys

**Figure 13:**
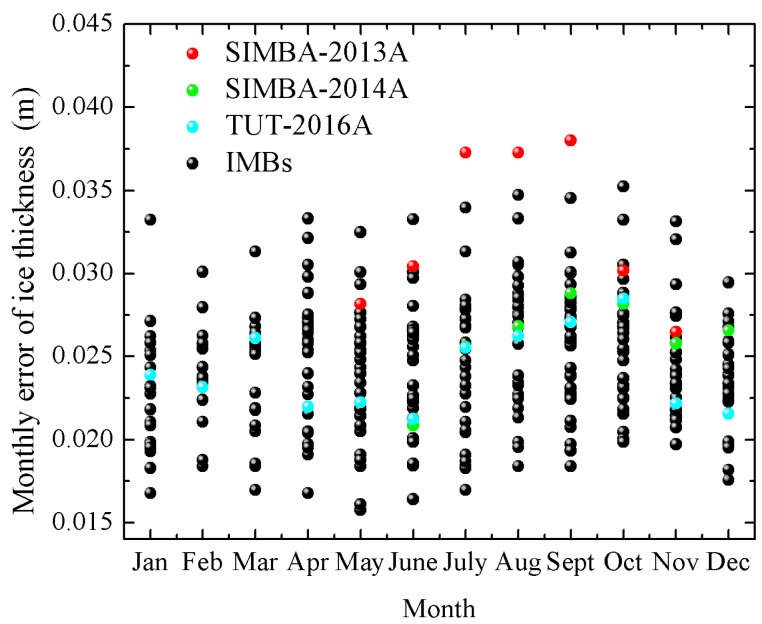
Mean monthly error values for buoys on Antarctic landfast ice and Arctic sea ice after processing with our amended analysis.

Data of temperature profiles measured on two sets of SIMBA deployed in Antarctica near Zhongshan Station in 2013, 2014 were used to discern ice thickness with algorithms. Because of regional variations in sea ice physical properties or variations in the external environment, such as air temperature, snowfall, and salinity, the applicability of the discrimination algorithm needs to be reassessed. In the same ice season of freezing period, for example, vertical temperature gradients of sea ice were different between Arctic and Antarctica. In Antarctica, the sea ice where SIMBA-2013A and SIMBA-2014A were deployed was first year ice with very thin snow depth (<0.10 m). Thus, the internal temperature of sea ice may be easily affected by air temperature changes, resulting in larger estimation errors. More measurements from temperature sensors or interpolated temperature were fitted to linear profile to calculate the intersection to improve the accuracy of calculating the ice thickness. As shown in Figure 13, we analyzed the monthly average sea ice thickness error of the buoys in the Arctic and Antarctica. This phenomenon may be attributed to the difference in vertical temperature gradients of sea ice.

## 6. Procedure to Determine Snow Depth and Ice Thickness

**Figure 14:**
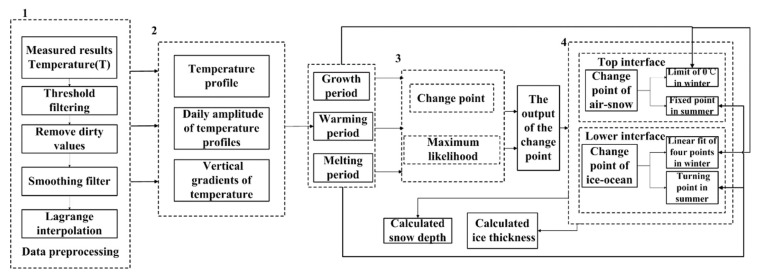
Schematic of the procedure for snow depth and ice thickness calculation during any period of ice season (ice growth, ice warming and ice melting) and deployment position (Arctic or Antarctica) by ice-tethered buoys. The bold numbers indicate major step in this approach, as noted in the paper.

To efficiently use the snow depth and ice thickness discrimination algorithm, a procedure of discrimination (Figure 14) was built. All sea ice temperature data must first go through the pretreatment process. We found that there were a lot of incomplete, inconsistent, and abnormal values in the original temperature profile data from the ice-tethered buoys, seriously affecting the efficiency of the algorithm. During data mining, the original data preprocessing process was also viewed as data cleaning. In general, data cleaning is mainly to delete irrelevant data or duplicate data, and to smooth the noise data in the original data set and filter out irrelevant data. In various thermistor strings of ice-tethered buoys, if the temperature-sensitive element is a thermal resistance type, such as platinum resistances or thermocouples, data loss in the temperature profile is more likely to occur than with semiconductor temperature sensors. We used interpolation to handle missing values by creating an interpolation function using a known point near the missing value to replace an unknown value; if the temperature value represents the temperature of the upper ocean, the fluctuations in the upper seawater temperatures are basically at a very small level, we used the average value of the neighboring values near the missing value as a substitute for this point.

The procedure to determine snow depth and ice thickness using the morphological temperature profile (linear or C–type) was according to the discussed determination modes. Information of ice season (growth period, warming period and melting period) were also considered. The top and lower interfaces were represented by change points to calculate snow depth and ice thickness. The discrimination of ice thickness was selected by maximum likelihood theory using the temperature profile. For the calculation of snow depth, change point theory was used to process temperature profile of sea ice.

**Figure 15:**
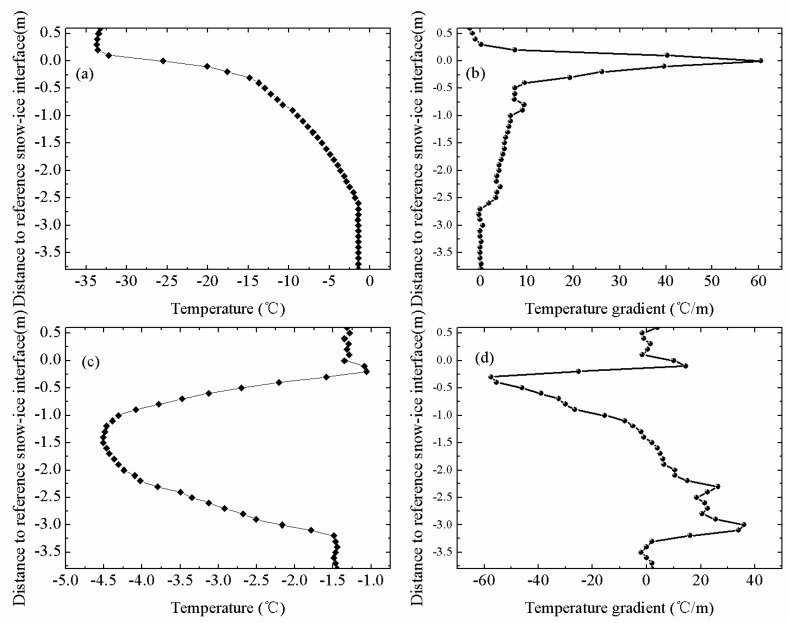
(**a**) Temperature profile of IMB-2007E in growth period. (**b**) The vertical gradients of temperature profile of IMB-2007E in growth period. (**c**) Temperature profile of IMB-2007E in warming period. (**d**) The vertical gradients of temperature profile of IMB-2007E in warming period. Two periods of sea ice can be identified based on the difference in shape between the temperature profiles and the vertical temperature gradients of two periods.

For any given temperature profiles of sea ice measured by ice-tethered buoys, the following discrimination algorithm procedure can be followed:

1. Data preprocessing. Firstly, measured results of temperature profile of sea ice are processed by threshold filtering and removing the outliers, such as 30 °C or −60 °C. In the second place, smoothing filter is used to filter out irrelevant data. In the third place, we use interpolation to handle missing values by creating an interpolation function to replace the null values of the temperature profile of sea ice.
2. Based on the temperature profiles, the vertical gradients of temperature profiles are calculated. Then the period of sea ice is determined.
  (1) Calculate the intersection of the temperature profile and 0 °C. If the intersection is lower than the initial snow-ice interface, the period of sea ice is the melting period.
  (2) If the intersection is higher than the initial snow-ice interface, the temperature profiles and the vertical gradients of temperature profiles are used to identify the period of sea ice. If the vertical temperature profile is linear, it is the ice growth period. If the temperature profile is C-type, it is the warming period. And in the growth and warming period, the vertical gradients of temperature profiles are different, thus the period can be identified as shown in Figure 15.
3. Use the change point theory and maximum likelihood theory to generate the output of the change points.
4. Use the change points to obtain the top and lower interfaces as described in Section 3.1. The snow depths and ice thicknesses are then obtained.

## 7. Conclusions

This research demonstrated an approach for calculating snow depth and ice thickness in the Arctic Ocean and Antarctic from temperature profiles, daily amplitude and vertical gradients of temperature profile observed by the ice-tethered buoys. We transformed the problem of calculation of snow depth and ice thickness into a change point detection problem, and evaluated the applicability of theories of change point and maximum likelihood in the determination of snow depth and ice thickness. A procedure was developed to use data of temperature profiles of sea ice to calculate the top and lower interfaces of the layer of snow-covered ice applicable to various ice seasons, temperature sensors interval, initial ice thickness, deployment position. The results indicated that calculated snow depth and ice thickness maintain acceptable range of error. Estimation ice thickness errors in summer were significantly larger than in winter. The higher resolution of the temperature measurement can effectively reduce the error in actual calculation of ice thickness. By analyzing the effect of initial ice thickness, the algorithm and procedure are more adaptable to rapidly changing Arctic sea ice because the smaller the initial ice thickness could be easier to build sea ice temperature gradient thus have smaller estimation errors for ice thickness and snow depth. The practicality of this procedure was examined. Results also indicated that the application of discrimination algorithm and procedure can effectively improves the scientific value of the data of temperature profile through the snow-covered ice even without the joint deployment of acoustic sensors to measure the ice thickness and snow depth.

The ice-tethered buoys used in this study are IMBs, SIMBAs and one TUT buoy. The SIMBAs have a heating mode that effectively distinguishes the snow-ice interface. Therefore, the combination of the data of heating mode of SIMBAs and our discrimination algorithm together could be further used to distinguish the snow-ice interface in the next step. In the current study, we don’t detail with the interface between snow and ice because the data measured by the SIMBAs is still limited. In addition, we plan to optimize observation instruments set up in the buoy. For instance, the TUT can be assembled with a thermistor string of 10 m or longer, which a sensor spacing is 0.02 m or smaller. The accuracy of the thermistor string can be increased to reach 0.01 °C by replacing the semiconductor temperature sensor with a more accurate platinum resistor to improve the accuracy of snow depth and ice thickness estimation. The sensor of conductivity and temperature (CT) can be assembled to measure the salinity and temperature of seawater just under the ice cover. In this study, 48 buoys deployed in the Arctic Ocean and two SIMBA buoys deployed in Antarctica were used. Therefore, in future work, we will strengthen the analysis of the data of the ice-tethered buoys deployed in Antarctica, especially for the ice zones further offshore, to enhance the applicability of our algorithm. Different ice types should be considered and analyzed when using our algorithm. For instance, estimated ice thickness should be combined with ice types such as thick or thin multiyear ice, hummock, ponded ice, deformed ice, and ice ridges to enhance applicability and reduce the uncertainties of the discrimination algorithm under different ice types.

## Figures and Tables

**Table 1:** Critical parameters of ice-tethered buoys.

Buoy Type	Interval of Temperature Sensor/m	Length of Thermistor String/m	Buoy Number	Sonar ^1^	Temperature Accuracy/°C
IMB	0.10	4.5	47	Y	±0.1
SIMBA	0.02	4.8	2	N	±0.1
TUT	0.03	4.5	1	Y	±0.1

^1^ The sonar was used for observation of snow depth or ice thickness. The SIMBAs had no sonars, thus the snow depth and ice thickness of the SIMBAs were measured by drilled-holes.

**Table 2:** Results of the two methods.

Method	Parameter	Error of Snow Depth/m	Error of Ice Thickness/m
Change point method	Temperature profile	0.036 ± 0.016	0.039 ± 0.017
Change point method	Daily amplitude of temperature	0.046 ± 0.016	
Change point method	Vertical gradients of temperature	0.046 ± 0.016	0.038 ± 0.017
Maximum likelihood method	Temperature profile	0.038 ± 0.015	0.036 ± 0.017
Maximum likelihood method	Daily amplitude of temperature	0.037 ± 0.016	
Maximum likelihood method	Vertical gradients of temperature	0.038 ± 0.016	0.037 ± 0.016
